# Public participation, institutional trust, and distributive justice in wind and solar projects

**DOI:** 10.1007/s00267-026-02520-2

**Published:** 2026-06-01

**Authors:** Tiago Miguel, Margarita Robaina, Anabela Botelho, Fátima Lima, Edimar Ramalho, Mara Madaleno

**Affiliations:** 1 DEGEIT & GOVCOPP, University of Aveiro, Campus Universitário de Santiago, Aveiro, Portugal; 2 CIICESI, Escola Superior de Tecnologia e Gestão, Politécnico do Porto, Rua do Curral, Casa do Curral, Felgueiras, Portugal

**Keywords:** Institutional trust, Distributive justice, Public participation, Social acceptance, Wind and solar energy projects

## Abstract

Scaling up wind and solar energy is essential for decarbonization, yet local contestation frequently delays projects, making social acceptance a critical enabling condition. This study examines the institutional drivers of acceptance by analyzing how trust, participation, transparency, and perceived impacts shape residents’ evaluations of renewable energy developments. It addresses four questions: how trust in key stakeholders influences perceptions of distributive justice; whether participation reduces perceived negative impacts and increases acceptance; whether these dynamics differ between wind and solar; and how energy literacy, information availability, and desired engagement relate to support. Using comparative evidence from 300 residents in the Centro Region of Portugal, the study models relationships among stakeholder‑specific trust, participation experiences, perceived impacts, and fairness evaluations. The findings show that trust, perceived fairness, and meaningful engagement are far more influential than procedural formality or technical knowledge. Trust in institutions strongly predicts perceived distributive justice and willingness to pay, while awareness of Environmental Impact Assessments or regulatory procedures has no measurable effect. Participation consistently aligns with more positive fairness perceptions, and perceived impacts remain a key determinant of support. The study contributes by identifying which institutions matter most for perceived fairness, demonstrating the limits of transparency understood as formal compliance, and clarifying when participation enhances legitimacy—offering guidance for more socially robust renewable energy governance.

## Introduction

Rapid expansion of wind and solar energy is essential for decarbonization, yet local controversy frequently delays projects, making social acceptance a central challenge in renewable energy transitions (Bourdin, 2026; IPCC, 2022; Wüstenhagen et al., 2007). Research shows that broad support for renewables often coexists with resistance to specific projects (Sun et al., 2025; Shen and Tai, 2024; Susskind et al., 2022), highlighting the importance of local governance contexts—how projects are planned, permitted, and managed (Enserink et al., 2022; Aitken, 2010). Institutional actors such as governments, regulators, developers, and civil society organizations play a decisive role in shaping conflict, legitimacy, and support (Schnell and Mattes, 2026; Radtke, 2025; Sovacool et al., 2022; Batel et al., 2013; Wolsink, 2007).

The social acceptance literature conceptualizes acceptance as multi‑dimensional, spanning socio‑political, community, and market acceptance (Xu et al., 2023; Delcayre and Bourdin, 2025; Al‑Emran, 2023; Wüstenhagen et al., 2007). Acceptance is institutional and relational, shaped by perceptions of decision‑making quality (Grelle and Hofmann, 2024), by the credibility of authorities and developers (Shah and Asghar, 2024; Knauf and Wüstenhagen, 2023; Ryder et al., 2023), and by the perceived fairness of outcomes (Modica and Rampa, 2026; Aitken, 2010). Trust—both in institutions and developers—is repeatedly identified as pivotal for legitimacy (Ellis and Ferraro, 2016; Aitken, 2010).

Energy justice provides a framework for linking trust to acceptance through distributive justice (how benefits and burdens are shared) and procedural justice (how decisions are made and who is heard) (Wang and Lo, 2023; Faulques et al., 2022; Ndi, 2024). Recognition—being respected and represented—is also central (Hoesch et al., 2025; Jenkins et al., 2016; Sovacool and Dworkin, 2015). In wind energy conflicts, unfairness intensifies opposition, while fair process increases legitimacy even when outcomes are contested (de Fine Licht and Håkansson, 2025; Ellis and Ferraro, 2016; Gross, 2007). However, symbolic or overly technical participation can undermine trust (Ndi, 2024; Ellis and Ferraro, 2016; Aitken, 2010). When participation is tokenistic (“symbolic”) or designed in a highly technical way that lay participants cannot realistically engage with, people perceive the process as unfair or manipulative, which reduces perceived procedural justice and transparency—and that, in turn, undermines trust in project developers and institutions.

Distributive justice is especially salient in just transitions, where debates focus on landscape impacts, who benefits financially, and whether compensation is fair (Hoesch et al., 2025; Jenkins et al., 2016; Sovacool and Dworkin, 2015; de Fine Licht and Håkansson, 2025; Ndi, 2024; Faulques et al., 2022; Gross, 2007; Wolsink, 2007). Because the distribution of benefits and burdens is managed through institutional decision-making processes (Shah and Asghar, 2024), residents’ trust in public authorities, NGOs, regulators, and media actors influences how they evaluate the fairness and legitimacy of renewable energy projects (Modica and Rampa, 2026; de Fine Licht and Håkansson, 2025; Ndi, 2024; Faulques et al., 2022; OECD, 2017; Ellis and Ferraro, 2016).

Portugal provides a particularly relevant context for examining renewable energy acceptance because the country has pursued ambitious decarbonization and renewable energy expansion targets while simultaneously facing growing local contestation surrounding the siting of wind and solar projects. Recent renewable energy deployment strategies have intensified pressure on rural and peri-urban territories, increasing the importance of procedural legitimacy, institutional trust, and distributive fairness in local governance processes. The Portuguese case, therefore, offers insight into how national energy transition objectives interact with community-level perceptions and governance practices during accelerated renewable energy deployment.

The literature has established that social acceptance of renewable energy is shaped by trust, participation, procedural and distributive justice, and transparency; however, less is known about how these factors interact across institutional and project-specific dimensions, particularly in wind and solar projects. Building on this gap, the present study examines how institutional trust, participation, distributive justice, and transparency jointly shape residents’ acceptance of renewable energy developments. This study examines how institutional and participatory factors shape the social acceptance of renewable energy projects. More specifically, it addresses two overarching questions:

RQ1: How do institutional trust, participation, and perceptions of distributive justice influence the social acceptance of wind and solar projects? (Grelle and Hofmann, 2024; Shah and Asghar, 2024; Knauf and Wüstenhagen, 2023; Ryder et al., 2023; OECD, 2017; Gross, 2007; Ellis and Ferraro, 2016; Aitken, 2010).

RQ2: To what extent do transparency, information availability, and technology-specific perceptions shape residents’ evaluations of renewable energy developments? (Wüstenhagen et al., 2007; Batel et al., 2013; Ehanmo, 2024; Ellis and Ferraro, 2016; Frederiks et al., 2015).

These broader questions are operationalized through a set of complementary hypotheses concerning participation, trust, distributive justice, willingness to pay, and governance preferences.

By integrating stakeholder‑specific trust, distributive justice, participation quality, transparency, and technology‑specific acceptance, the study offers a more granular understanding of how communities evaluate the “justness” of renewable energy deployment (Jenkins et al., 2016; Wüstenhagen et al., 2007). It identifies which institutional relationships matter most for fairness perceptions, how participation quality shapes acceptance, how wind and solar differ in governance evaluations, and how information and engagement preferences influence responses to new investments.

This paper makes four original contributions. First, it empirically disaggregates trust by stakeholder type—government, regulator, developers, NGOs, and the media—and tests how each channel is associated with perceived distributive justice and acceptance, moving beyond single “institutional trust” measures. Second, it distinguishes between participation quantity and participation quality by examining participation and transparency as governance attributes that can either build or erode legitimacy, clarifying when engagement is perceived as meaningful rather than symbolic or overly technical. Third, it provides a technology-comparative perspective, identifying where governance evaluations and acceptance mechanisms diverge between wind and solar projects, and how these patterns interact with energy literacy and information availability. Finally, a central contribution of the study is the analytical distinction between meaningful participation and procedural formality, showing that formal compliance with regulatory procedures does not necessarily translate into perceived legitimacy, trust, or social acceptance. Together, these advances connect energy justice and social acceptance with actionable evidence on which institutional relationships and governance practices most strongly shape locally legitimate renewable deployment.

## Literature Review

### Public consultation and perception of fairness

The literature on renewable energy governance uses terms such as “social acceptance,” “public acceptance,” and “acceptance” in partially overlapping ways (Wüstenhagen et al., 2007). In this study, social acceptance is used as the broader analytical concept referring to the societal legitimacy and support associated with renewable energy technologies, projects, and governance arrangements. Public acceptance is employed more specifically to describe the attitudes, perceptions, and evaluations expressed by residents and local communities affected by renewable energy developments. The term acceptance is used only as a shorthand expression where the context clearly refers to these broader dimensions. Clarifying these distinctions is important because support for renewable energy technologies at a general societal level does not necessarily imply acceptance of specific projects, locations, or governance processes.

Research on renewable energy “social acceptance” emphasizes that acceptance is not only about attitudes toward technologies, but also about confidence in institutions, perceived fairness, and the quality of governance processes surrounding siting and permitting (Modica and Rampa, 2026; Hoesch et al., 2025; Ndi, 2024; Batel et al., 2013; Aitken, 2010; Wüstenhagen et al., 2007). The multi-dimensional framing of acceptance—socio-political, community, and market acceptance—helps to connect local project support to institutional arrangements and actor relationships (Modica and Rampa, 2026; Wüstenhagen et al., 2007). Within community acceptance in particular, justice (procedural and distributive) and trust are consistently identified as central drivers of support or opposition to wind (and, increasingly, solar) developments (Modica and Rampa, 2026; Ellis and Ferraro, 2016; Gross, 2007; Wolsink, 2007).

Energy justice literature provides concepts that map directly onto project-level controversies: distributive justice (who gets benefits and who bears burdens), procedural justice (how decisions are made and who can influence them), and recognition (who is respected and represented) (Jenkins et al., 2016; Sovacool and Dworkin, 2015). These studies jointly motivate hypotheses linking consultation, transparency, and trust to perceived fairness and support for projects (Enserink et al., 2022).

A large body of work argues that procedurally fair decision-making can shape perceptions of legitimacy and acceptance of energy infrastructure (Bourdin, 2026; Susskind et al., 2022). In wind energy conflicts, a “fair process effect” has been documented: when residents perceive decision processes as fair, they may judge outcomes as more legitimate, even if they dislike the outcome (Knauf and Wüstenhagen, 2023; Gross, 2007). This aligns with broader procedural justice research showing that opportunities for public voice, respectful treatment, and residents’ perceptions that decision-makers act impartially can increase acceptance of decisions—even unpopular ones—by strengthening perceived legitimacy (Grelle and Hofmann, 2024; Ellis and Ferraro, 2016; Gross, 2007).

However, the literature warns that consultation only helps if it is genuine and gives people real influence (Bourdin, 2026; Delcayre and Bourdin, 2025). If engagement is perceived as symbolic, “done deal,” or controlled by developers/authorities, it can erode trust and heighten conflict rather than resolve it (Bourdin, 2026; Delcayre and Bourdin, 2025; Ellis and Ferraro, 2016; Aitken, 2010). This supports studying participation quality and perceived responsiveness as mediators of whether participation actually increases perceptions that concerns were addressed.

Public consultation is also connected to perceptions of distributive justice because benefit sharing and compensation are frequently negotiated, explained, or contested within participatory forums. In practice, the perceived fairness of community benefit arrangements depends not only on the amounts but also on how decisions about benefits are made, who is eligible, and whether the process is transparent and inclusive (Sun et al., 2025; Susskind et al., 2022; Aitken, 2010; Gross, 2007). Community energy and local governance research similarly distinguishes “process” (degree and quality of involvement) and “outcomes” (distribution of benefits) and highlights that local involvement can change how distributive outcomes are interpreted and accepted (Shen and Tai, 2024; Enserink et al., 2022; Walker and Devine-Wright, 2008).

With this in mind, the first set of testable hypotheses is formulated. For H1: Public consultation and perception of fairness.

*H1a: Greater participation in public consultation is positively associated with the perception that project-related concerns have been adequately addressed*.

*H1b: Greater participation in public consultation is positively associated with the perception that the distribution of project benefits is fair*.

### Transparency and Information

Transparency and information are repeatedly discussed as prerequisites for legitimate environmental decision-making, especially where perceived risks and local impacts are salient. In disputes over wind projects, distrust tends to grow when people feel information is hidden, too technical to understand, or presented in a biased way. On the other hand, being open and clear can strengthen legitimacy by allowing people to check the facts and feel less suspicious (Bourdin, 2026; Shen and Tai, 2024; Sovacool et al., 2022; Ellis and Ferraro, 2016; Aitken, 2010). Since Environmental Impact Assessment (EIA) is a formal governance mechanism intended to identify, communicate, and mitigate impacts, awareness that an EIA exists may function as a signal that due process and oversight are in place—potentially increasing perceived procedural fairness and confidence in impact management (Schnell and Mattes, 2026; Xu et al., 2023; Enserink et al., 2022; Ellis and Ferraro, 2016; Wolsink, 2007). That said, the same literature warns that formal processes do not automatically generate legitimacy if they are viewed as box-ticking or inaccessible (Radtke, 2025; Ellis and Ferraro, 2016; Aitken, 2010).

Energy literacy links to support through several mechanisms: understanding technologies and trade-offs can reduce reliance on misinformation, increase perceived efficacy of renewables, and improve engagement capacity (Bourdin, 2026; Delcayre and Bourdin, 2025; Al-Emran, 2023). Behavioral and energy decision-making research shows that knowledge is related to (but not sufficient for) pro-energy-transition behaviors, and that cognitive constraints and informational environments shape choices and acceptance (Ehanmo, 2024; Cheng and Lee, 2022; Frederiks et al., 2015). In renewable acceptance contexts, education and information are often included as relevant correlates of attitudes, even as scholars caution against simplistic “deficit model” assumptions (i.e., that opposition is merely ignorance) (Bourdin, 2026; Delcayre and Bourdin, 2025; Al-Emran, 2023; Aitken, 2010).

Therefore, the second set of hypotheses related to H2: Transparency and information are going to be tested.

*H2a:* Greater awareness of the Environmental Impact Assessment (EIA) process is positively associated with acceptance of renewable energy projects.

*H2b:* Higher energy literacy is positively associated with support for new wind and solar energy projects.

### Institutional Trust

Trust is a central theme across research on renewable energy acceptance. Critics of narrow NIMBY (“Not In My Backyard”) framings argue that opposition often reflects concerns about governance, fairness, and whether institutions and developers are acting competently and honestly (Modica and Rampa, 2026; Xu et al., 2023; Batel et al., 2013; Aitken, 2010). Trust matters for acceptance because people must rely on what others say about impacts, how harms will be reduced, and whether promised benefits will actually happen. Given the uncertainty, the messenger’s credibility becomes very important (Bourdin, 2026; Schnell and Mattes, 2026; Savacool et al., 2022; Ellis and Ferraro, 2016). Institutional trust frameworks in public policy also identify perceived fairness, openness, and reliability as core drivers of trust in public institutions (OECD, 2017).

In stated-preference studies, “protest zeros” (Willingness to pay (WTP) = 0, not because the value is truly zero, but because of objections to institutions, processes, payment vehicles, or perceived unfairness) are widely recognized in contingent valuation and discrete-choice contexts. Distrust in implementing institutions or objection to governance is a classic driver of refusal to pay in principle, even when respondents may support the underlying good (e.g., environmental improvement) (Sun et al., 2025; Enserink et al., 2022; Arrow et al., 1993). Based on the stated-preference literature on protest responses, distrust and objections to governance/benefit allocation may increase protest zero WTP (Susskind et al., 2022; Xu et al., 2023; Mitchell and Carson, 1981, 1989). In renewable project contexts, distrust may similarly increase the likelihood of WTP = 0 as a form of protest against decision-makers or benefit allocation (Grelle and Hofmann, 2024; Knauf and Wüstenhagen, 2023).

WTP is interpreted in this study not only as an economic valuation measure, but also as an indicator of institutional legitimacy and support for renewable energy governance arrangements. In this context, zero willingness to pay may reflect financial limitations. However, it may also constitute a form of protest response rooted in distrust, perceived unfairness, or opposition to the institutional arrangements associated with project implementation.

Having this literature in mind, the third set of hypotheses is formulated, related to H3: Institutional confidence.

*H3a:* Greater trust in decision-makers is positively associated with support for new renewable energy projects.

*H3b:* Greater distrust in decision-makers is positively associated with a higher likelihood of zero willingness to pay.

In this study, institutional trust refers to the perceived credibility, competence, fairness, and reliability of the actors involved in renewable energy governance, including public authorities, regulators, developers, environmental organizations, and media actors. Trust is treated not simply as general political confidence, but as a project-related evaluative judgment shaping whether residents believe institutions will manage impacts fairly, communicate honestly, and implement projects responsibly.

### Stakeholder involvement

Participation and governance are multi-actor processes in renewable deployment, involving developers, regulators, multiple government levels, NGOs, and the media as information intermediaries (Grelle and Hofmann, 2024; Batel et al., 2013; Wüstenhagen et al., 2007). When trust is uneven across actors, publics may prefer decision authority and oversight to sit with the actors they view as more competent, fair, or accountable (Knauf and Wüstenhagen, 2023; Ryder et al., 2023). Public trust research distinguishes trust across institutions and levels of government and suggests that perceived responsiveness and fairness influence these trust judgments (Shah and Asghar, 2024; OECD, 2017). Translating this to siting governance, people who distrust central government may prefer stronger municipal involvement because local authorities can be perceived as more accessible, place-sensitive, and accountable (OECD, 2017; Wolsink, 2007).

Thus, the final set of testable hypotheses is formulated, regarding H4: Stakeholder involvement.

*H4a:* Greater institutional trust is positively associated with support for broader stakeholder involvement in decision-making.

*H4b:* Greater distrust in the central government is positively associated with a preference for stronger municipal involvement in decision-making.

Despite strong agreement that procedural fairness, transparency, and trust matter for the acceptance of renewable energy, the literature leaves several gaps that the present study will address. First, much of the empirical evidence on the “fair process effect” and meaningful participation comes from wind cases, while solar is less systematically examined, making it unclear whether participation has similar effects across technologies and siting contexts (Modica and Rampa, 2026; Ellis and Ferraro, 2016; Aitken, 2010; Gross, 2007). Second, although transparency is often mentioned, fewer studies measure it using clear, policy-relevant indicators—like whether people know an EIA was carried out—and then test if that knowledge is linked to acceptance on its own, separate from general trust and perceptions of fairness (Faulques et al., 2022; Ellis and Ferraro, 2016; Wolsink, 2007). Third, although distrust is central to acceptance debates, it is less often linked to economic-preference outcomes; bringing in stated-preference insights into protest responses allows this study to test whether distrust increases the likelihood of WTP = 0 (Hoesch et al., 2025; Ndi, 2024; Arrow et al., 1993; Mitchell and Carson, 1981, 1989). Finally, work on multi-actor governance highlights that the publics differ in their perceptions of institutions. However, there is limited hypothesis-driven evidence linking trust profiles to preferences for which stakeholders should be most involved—particularly whether distrust in the central government maps onto stronger preferences for municipal involvement (Grelle and Hofmann, 2024; Knauf and Wüstenhagen, 2023; Ryder et al., 2023; OECD, 2017; Wolsink, 2007; Wüstenhagen et al., 2007). By jointly modeling participation, information/EIA awareness, energy literacy, institutional trust, and stakeholder involvement preferences for both wind and solar projects, this study tests an integrated institutional pathway to distributive justice perceptions and support, as the existing literature motivates but rarely evaluates in a single comparative framework.

### Procedural Formality and Meaningful Participation

The literature on procedural justice and renewable energy governance increasingly distinguishes between formal procedural compliance and meaningful public participation (Walker and Devine-Wright, 2008; Gross, 2007). In this study, procedural formality refers to the existence of institutionalized and legally mandated administrative procedures, including Environmental Impact Assessments (EIAs), licensing processes, disclosure obligations, and officially required public consultations. These mechanisms are designed to ensure regulatory compliance and procedural transparency within project approval processes.

However, the existence of formal procedures does not necessarily imply that local communities perceive decision-making processes as fair, inclusive, or influential. Meaningful participation instead refers to the extent to which affected residents believe they can express concerns, access understandable information, influence outcomes, and receive respectful treatment during project development processes. Procedural justice research has shown that perceptions of legitimacy depend not only on whether participatory mechanisms formally exist, but also on whether individuals perceive these mechanisms as substantively responsive and capable of influencing decisions (Smith and McDonough, 2001).

This distinction is particularly relevant in renewable energy governance, where consultation procedures are frequently implemented as part of regulatory compliance. However, it may still be experienced by local communities as symbolic, inaccessible, or ineffective. The study, therefore, distinguishes analytically between procedural formality and meaningful participation to evaluate whether formal institutional compliance alone is sufficient to generate social acceptance and institutional legitimacy.

### Conceptual Distinctions: Participation, Procedural Formality and Institutional Trust

This study distinguishes analytically between participation, procedural formality, and regulatory procedures, although these concepts are related in practice (OECD, 2017; Brownsword and Goodwin, 2012). Participation refers to residents’ perceived opportunities to express concerns, influence decisions, and engage meaningfully with project development processes, emphasizing the experiential and relational dimensions of governance, particularly whether individuals feel heard, respected, and capable of influencing outcomes (OECD, 2017). Procedural formality, by contrast, refers to the existence of formal institutional mechanisms and legally required administrative procedures, such as Environmental Impact Assessments (EIAs), licensing processes, disclosure requirements, or officially mandated public consultations; these mechanisms represent institutional compliance with regulatory obligations but do not necessarily imply meaningful public influence or perceived legitimacy (UNECE, 2006; Brownsword and Goodwin, 2012). Regulatory procedures constitute the broader institutional framework within which participation may occur. While procedures such as EIAs often contain participatory components, awareness of these procedures does not necessarily translate into perceptions of inclusion, fairness, or trust (UNECE, 2006; OECD, 2024). This distinction is central to the paper’s analytical framework because the study evaluates whether formal procedural compliance alone is sufficient to generate social acceptance, or whether acceptance depends more strongly on relational dimensions of governance such as trust, responsiveness, and meaningful engagement (OECD, 2017; OECD, 2024).

## Data and methodology

This study adopts a cross-sectional comparative survey design to examine how institutional trust, participation, perceptions of fairness, and transparency shape the social acceptance of renewable energy projects. The research combines descriptive and econometric analyses to evaluate relationships between governance-related perceptions and support for wind and solar developments.

Rather than examining isolated determinants of renewable energy acceptance, the study adopts an integrated institutional framework linking participation, transparency, trust, distributive justice, and support for renewable projects. The study combines descriptive cross-tabulations with Probit, Tobit, and Ordered Probit models because it examines multiple interconnected dimensions of social acceptance, each with distinct dependent variables. Descriptive analyses were particularly important in cases with limited statistical power. At the same time, the econometric models allowed the study to test relationships between governance-related variables and acceptance indicators under different outcome structures. Together, these approaches provide complementary evaluations of the institutional, procedural, and distributive dimensions of renewable energy acceptance.

The study surveyed approximately 300 adult residents in the Centro Region of Portugal, selected from municipalities with high wind and solar deployment relevance. Eligibility criteria were minimal, participation was voluntary, and data were fully anonymized. All questionnaire-based procedures complied with applicable ethical and data-protection requirements. Prior to fieldwork, the full study protocol (research objectives, sampling and recruitment procedures, questionnaire and consent script, data management plan, and risk assessment) was submitted to and approved by the University of Aveiro’s Ethics Committee. The study was conducted in accordance with the General Data Protection Regulation (GDPR) and relevant national implementing legislation. Respondents received clear written/oral information about the study purpose, what participation involved, expected duration, potential risks and benefits, the absence of penalties for non-participation, and their right to withdraw at any time without providing a reason. Informed consent was obtained before the questionnaire started. No sensitive personal data were collected; where socio-demographic variables were required for analysis, they were limited to categories necessary for the research questions and were recorded in a way that minimized identifiability.

Questionnaires were distributed across key municipalities representing different exposure contexts. Among the most populous municipalities, Torres Vedras (wind case) contributed 68 responses; Ovar (solar case) contributed 45; and Águeda (no‑park comparison case) contributed 37. Together, these municipalities account for roughly half of the total sample and provide variation in local experience with renewable energy projects.

In the less populous municipalities of the Centro Region, Castro Daire (wind case), Alcanena (solar case), and Santa Comba Dão (no‑park comparison) contributed 43, 58, and 49 questionnaires, respectively, totaling 150 responses. This distribution ensures variation in local experience with wind and solar projects while maintaining balanced representation across municipalities. Municipalities were selected purposively to ensure variation in local exposure to renewable energy infrastructures, institutional experiences, and community familiarity with project development processes, including municipalities associated primarily with wind projects, solar projects, and comparison municipalities without major renewable energy parks, which allowed the study to explore whether governance perceptions and acceptance dynamics differed across technological and territorial contexts.

Data were collected through structured questionnaires administered to adult residents across the selected municipalities. Questionnaires were distributed mainly online, with a combination of in-person and local community-based dissemination approaches to maximize participation across different demographic groups. Participation was voluntary and anonymous, and respondents were informed of the study’s academic purpose prior to participation. The questionnaire included sections on institutional trust, perceptions of fairness, participation experiences, attitudes toward renewable energy, willingness to pay, and preferences for stakeholder involvement.

The sociodemographic profile is broadly diverse, with a near‑balanced gender distribution (164 women (54.7%) and 136 men (45.3%)). and a mix of marital statuses. These characteristics provide useful context for interpreting environmental perceptions, as prior research suggests that gender and household roles can shape attitudes and practices related to energy and sustainability (e.g., Zelezny et al., 2000; Kennedy and Kmec, 2018). Some survey modules—particularly the technology‑specific items—were completed by fewer respondents (116 for wind‑specific items; 108 for solar), resulting in partial non‑response that limits the depth of certain comparisons.

The study addresses two research questions already mentioned, concerning: RQ1: How do institutional trust, participation, and perceptions of distributive justice influence the social acceptance of wind and solar projects?; and RQ2: To what extent do transparency, information availability, and technology-specific perceptions shape residents’ evaluations of renewable energy developments? Table 7 summarizes all variables, hypotheses, and analytical strategies.

The use of multiple econometric specifications reflects the multidimensional nature of social acceptance. Because the study examines different but interconnected dimensions of acceptance—including procedural fairness, distributive justice, willingness to pay, and governance preferences—different model specifications are required to match the structure of each dependent variable appropriately. Together, these models provide a coherent evaluation of the institutional determinants of renewable energy acceptance.

Given the structure of the dependent variables—binary outcomes, censored monetary values, and ordered involvement scales—the analysis employs Probit, Tobit, and Ordered Probit models. Robust and clustered standard errors by municipality strengthen inference by accounting for heteroskedasticity and intra‑municipal correlation. This alignment between hypotheses, data structure, and modeling strategy ensures that each research question is addressed with an appropriate, statistically sound method.

The Probit models used for H1a, H1b, H2a, and part of H2b are appropriate because these hypotheses involve binary outcomes, such as whether concerns were considered or whether respondents perceived benefits or negative impacts. These variables reflect latent attitudes expressed as yes/no responses, and Probit models estimate the probability of such outcomes under assumptions consistent with social‑perception processes. This allows the analysis to quantify how participation, transparency, or energy literacy shape the likelihood of acceptance or perceived fairness.

The Tobit models applied to H3a, H3b, and the willingness‑to‑pay items address the fact that the monetary contribution variable is censored at zero. Many respondents report zero legitimately—often as a form of institutional or moral rejection—so treating these values as continuous would bias estimates. Tobit models capture both the decision to contribute and the amount contributed, enabling tests of how trust, perceived benefits, environmental concerns, and financial constraints influence WTP.

The Ordered Probit models used for H4a and H4b match the ordinal structure of desired stakeholder involvement, which ranges from “no involvement” to “a lot of involvement.” These categories have a clear ranking but no numeric distance. Ordered Probit models preserve this structure and estimate how trust or distrust in different actors shapes preferences for their involvement in project governance, consistent with theories of graded governance preferences.

Several methodological limitations should be acknowledged. First, the study focuses on a limited number of municipalities within the Centro Region of Portugal, which may restrict the generalizability of the findings to other regional or national contexts with different socio-political, economic, or institutional characteristics. Second, the cross-sectional survey design captures perceptions at a single point in time and therefore does not allow for causal inference or analysis of how attitudes evolve over the course of project development. In addition, some technology-specific analyses rely on relatively small subsamples due to partial non-response, reducing statistical power in certain estimations. The study should therefore be interpreted primarily as an exploratory and comparative analysis of institutional dimensions of renewable energy acceptance.

## Empirical results and discussions

### Public Consultation and Perception of Justice

Models 1–4 were estimated using Probit regressions to test H1a, H1b, and H2a. Due to substantial non‑response, the statistical power of these models is limited, and the estimates do not show significant effects of participation, perceived fairness, or EIA awareness on acceptance or perceived impacts. This directly affects the evaluation of Research Questions 2 and 3. The descriptive cross‑tabulations (Table 1), however, provide a clearer and more reliable picture of the relationships underlying H1a and H1b. Across all three tables, participation is consistently associated with more positive evaluations of procedural and distributive fairness.

**Table 1:** Cross-tabulations among variables of interest (complement of Table 8 estimations)

	You feel that your concerns and interests have been considered during the development of your municipality’s park(s)?			
	Participated in the public consultation process?	No	Yes	Total
	No	7	13	20
	Yes	2	10	12
	Total	9	23	32
	Have the perception that there are financial benefits for the municipality or the local community?			
Participated in the public consultation process?	No	Yes	I do not know	Total
No	2	13	5	20
Yes	2	10	0	12
Total	4	23	5	32
	Do you feel that the distribution of these benefits is fair for the community?			
Participated in the public consultation process?	No	Yes	I do not know	Total
No	3	9	1	13
Yes	0	9	1	10
Total	3	18	2	23

Participants are far more likely to report that their concerns were considered, whereas only a small minority of non‑participants share this view. This pattern supports H1a by indicating that participation enhances perceptions of procedural responsiveness. Participants are also much more likely to perceive financial benefits for the municipality or community. The contrast with non‑participants is substantial, offering strong descriptive support for H1b. None of the non‑participants view the distribution of benefits as fair, while half of the participants do. This reinforces the conclusion that participation is linked to more favorable distributive‑justice perceptions.

Taken together, although the Probit models do not yield statistically significant coefficients, the descriptive evidence consistently aligns with H1a and H1b: participation is associated with more positive perceptions of both procedural and distributive fairness.

The first set of Probit models (Table 8) tests whether perceptions of procedural or distributive fairness predict participation in consultation. None of the coefficients for concerns‑addressed or fair‑benefits variables are statistically significant, and several are estimated as zero. This indicates that there is no detectable relationship between participation and perceptions of fairness in this dataset. As a result, the models do not support H1a or H1b, which expected that expected participation would be associated with more positive procedural or distributive evaluations.

The models examining whether participation reduces perceived negative impacts (for wind or solar) also show no significant effects. Participation does not appear to influence how residents evaluate project impacts or acceptance levels. This directly answers RQ1: Based on the available data, participation does not reduce negative perceptions or increase acceptance. However, this result is strongly conditioned by the limited number of valid observations.

The solar‑specific models (F38SN, F39SN) produce large and statistically significant coefficients, while the wind‑specific variables do not. Although the extremely small sample size (*N* = 16) limits reliability, the contrast suggests that perceptions of solar parks may evoke stronger reactions—positive or negative—than perceptions of wind farms. This relates to RQ2: while no differences emerge for transparency‑related variables, the results hint that experiential or emotional responses to solar installations may be more pronounced.

### Transparency and Information

The final model testing H2a includes only two observations, making it impossible to draw meaningful statistical conclusions (Table 8). All coefficients are zero or non‑significant, so H2a cannot be evaluated with the available data. When the Probit estimates and the descriptive cross‑tabulations are considered together, they point to the same substantive conclusion: knowledge of an Environmental Impact Assessment does not appear to reduce perceived negative impacts or increase acceptance of wind or solar parks. The lack of statistical significance reflects the extremely limited number of valid responses. At the same time, the descriptive tables show no clear differences between those who know an EIA was conducted and those who do not. Overall, the evidence indicates that EIA awareness does not drive acceptance in this dataset.

The cross‑tabulations in Tables 2 and 3 show no meaningful differences between respondents who know an EIA was conducted and those who do not. For wind energy, 38% of respondents without EIA knowledge perceive negative impacts, compared to 39% among those aware of the EIA; the pattern for solar is similarly flat. This indicates that EIA awareness does not reduce perceived risks.

**Table 2:** Cross tabulations of variables to explore Model 3 (view Table 7; complements Table 8)

	Do you believe that wind energy can generate negative impacts?		
Do you know if the park(s) project in your municipality has had an environmental impact assessment?	No	Yes	Total
No	88	43	131
Yes	54	27	81
Total	142	70	212
	Do you believe that solar energy can generate negative impacts?		
Do you know if the park(s) project in your municipality has had an environmental impact assessment?	No	Yes	Total
No	94	37	131
Yes	54	27	81
Total	148	64	212

Additional cross‑tabulations (Table 3) confirm that knowing about the EIA is not associated with greater familiarity with the park, lower discomfort, or stronger perceptions of benefits. The distributions are nearly identical across groups, suggesting that EIA knowledge does not shape how residents experience or evaluate the project.

**Table 3:** Cross tabulations of variables to explore Model 4 (continued) (view Table 7; complements Table 8)

Do you know if the park(s) project in your municipality has had an environmental impact assessment (EIA)?				
		No	Yes	Total
Do you know the wind farm located in your area?	No	41	13	54
	Yes	42	20	62
	Total	83	33	116
Do you feel uncomfortable with the presence of the wind farm?	No	74	31	105
	Yes	9	2	11
	Total	83	33	116
Do you feel that the wind farm brings benefits?	No	46	11	57
	Yes	37	22	59
	Total	83	33	116
Do you know if the park(s) project in your municipality has had an environmental impact assessment?				
		Não	Sim	Total
Do you know of a solar park in your area?	No	36	18	54
	Yes	20	34	54
	Total	56	52	108
Do you feel uncomfortable with the presence of the solar park?	No	53	44	97
	Yes	3	8	11
	Total	56	52	108
Do you feel the solar park benefits you?	No	44	28	72
	Yes	12	24	36
	Total	56	52	108

The Probit model for H2a in Table 8 cannot detect any effects due to the extremely small number of valid observations (*N* = 2). However, the descriptive evidence from Tables 2 and 3—based on much larger samples—shows the same pattern: there is no relationship between EIA knowledge and acceptance indicators. This means the null results in Table 8 reflect a genuine absence of association, not a statistical artifact.

Taken together, the evidence does not support H2a. Awareness of an EIA does not improve acceptance or reduce perceived negative impacts. This suggests that while EIAs are essential regulatory tools, their existence alone does not build trust or reassurance unless they are actively communicated and integrated into broader engagement processes.

The Tobit estimations for H2b and H3a (Table 4) reinforce this broader pattern: formal, technical knowledge has no measurable effect on acceptance, whereas experiential perceptions—especially perceived impacts—show a clearer, though modest, influence.

**Table 4:** Models 5 and 6 (6a and 6b) results: Tobit results (Table 7 complements)

	(1)	(2)	(3)	(4)	(5)	(6)
	GF47VMDP	GF47VMDP	GF47VMDP	GF47VMDP	GF47VMDP	GF47VMDP
G48PMI	–1.621	–1.621				
	(2.296)	(1.890)				
C10SN	–2.421	–2.421				
	(47.89)	(32.48)				
C9N	6.493	6.493				
	(4.418)	(4.732)				
C9H	–6.615	–6.615				
	(6.949)	(7.146)				
C9C	–3.211	–3.211				
	(4.829)	(7.217)				
C9GN	0.805	0.805				
	(3.970)	(6.227)				
C9E	35.65	35.65				
	(29.87)	(34.76)				
C9S	23.30	23.30				
	(19.01)	(16.49)				
C9GEO	–20.82	–20.82				
	(14.63)	(13.70)				
C9B	12.44	12.44^*^				
	(9.159)	(6.753)				
C9G	–6.207	–6.207				
	(6.838)	(5.812)				
C9EO	–16.96	–16.96				
	(15.59)	(21.50)				
C101I	–17.88^*^	–17.88^**^				
	(9.924)	(8.557)				
C101NPE	20.43	20.43				
	(15.62)	(19.13)				
C101RC	–3.722	–3.722				
	(11.27)	(10.21)				
C101FE	19.19	19.19				
	(14.02)	(12.83)				
C101RDE	–20.45	–20.45				
	(14.15)	(14.60)				
D20GL			3.188	3.188		
			(4.933)	(7.465)		
D20CS			–2.875	–2.875		
			(3.136)	(4.067)		
D20GC			–2.947	–2.947		
			(4.483)	(6.133)		
D20ERSE			6.947	6.947		
			(6.052)	(7.339)		
D20AA			–6.591	–6.591		
			(4.206)	(4.532)		
D20E			3.799	3.799		
			(2.794)	(3.340)		
G49RE					4.849	4.849
					(7.470)	(7.561)
G49RPE					–8.726	–8.726^*^
					(5.637)	(4.663)
G49IP					–19.63	–19.63
					(13.73)	(16.50)
G49IFF					26.03	26.03
					(23.79)	(24.24)
G49IE					–12.77	–12.77
					(16.11)	(14.45)
G49R					9.319	9.319
					(10.76)	(10.64)
var(e.GF47VMDP)	4395.6^**^	4395.6^**^	3181.1^**^	3181.1^**^	5886.2^**^	5886.2^**^
	(2067.1)	(1774.8)	(1586.4)	(1231.3)	(2863.7)	(2622.0)
N	199	199	276	276	300	300
Log-likelihood	–817.1	–817.1	–1086.8	–1086.8	–1250.1	–1250.1
Pseudo R2	0.0357	0.0357	0.00258	0.00258	0.00462	0.00462
AIC	1670.3	1644.3	2187.7	2183.7	2514.2	2510.2
BIC	1729.6	1660.8	2213.0	2201.8	2540.1	2528.7

Standard errors in parentheses. * *p* < 0.10, ** *p* < 0.05, *** *p* < 0.01. See Table 7 for acronyms. Columns (1), (3), (5), and (7) are for robust standard errors. Columns (2), (4), (6), and (8) correspond to clustered robust standard errors. The clustered variable consists of H73CR (What is your municipality of residence? Águeda; Alcanena; Castro Daire; Ovar; Santa Comba Dão; Torres Vedras)

The models testing H2b include several indicators of residents’ knowledge of environmental procedures, regulatory bodies, and licensing processes (view Table 4). Across all specifications, these variables remain statistically insignificant, with small and unstable coefficients and very low pseudo‑R² values. This shows that knowing which agency licenses the project, who regulates it, or what procedures exist does not influence acceptance levels. These results mirror the findings for H2a: procedural formality and technical knowledge do not translate into more positive perceptions. Such information remains abstract and disconnected from residents’ lived experience of the project and therefore does not shape their attitudes.

### Institutional Trust

The results for H3a (Table 4, columns 3–6) show that perceived impacts have a modest but tangible effect on acceptance. Among the various attitudinal variables, only C101I—perceived negative impacts—displays a consistent, statistically significant coefficient. Its negative sign indicates that residents who believe renewable energy projects generate strong negative impacts report lower acceptance. A second variable, C9B (attitudes toward biomass), shows a weak and inconsistent positive association. Overall, these findings offer partial support for H3a: perceived impacts matter, but general attitudes toward different renewable technologies do not reliably predict acceptance.

Models 7 and 8 (Table 7) could not be estimated due to insufficient data for the Tobit analysis. To complement the analysis of H3a, H3b, and RQ1, cross‑tabulations are therefore used. These results reinforce the broader empirical pattern: acceptance is shaped far more by trust, fairness, and perceived impacts than by technical knowledge or procedural awareness. The absence of Tobit estimates does not weaken this conclusion; the descriptive evidence provides a clear picture, especially regarding the role of trust.

H3b proposed that distrust increases the likelihood of reporting WTP = 0. The cross‑tabulation of justifications for zero willingness to pay (GF47VMDP) strongly supports this hypothesis. Forty‑one respondents selected G50NTC (“I have no confidence in the entities responsible for implementing the projects”), and all 41 reported a WTP of zero. There is no case in which distrustful respondents are willing to pay a positive amount. This provides compelling evidence that distrust produces categorical refusal, not merely lower levels of support.

Distrust emerges as a clear and powerful driver of refusal. The cross‑tabulations show that whenever respondents explicitly cite a lack of confidence in the responsible entities (G50NTC), their willingness to pay is always zero. Although the number of such cases is small, the pattern is unequivocal: institutional distrust is associated exclusively with total financial refusal, not merely lower contributions.

The open‑ended justifications reinforce this interpretation. Respondents frequently frame their refusal in terms of institutional and ideological objections: distrust in the State or local authorities (“there is a lot of money badly spent”, “I do not trust the responsible entities”), criticism of public management (“taxes should already cover this”, “the State is inefficient”), and rejection of individual financial responsibility (“I should not be the one to pay”, “companies should contribute”). These statements show that zero WTP is not primarily an economic constraint, but a principled stance rooted in distrust and perceived institutional failure. The refusal to contribute reflects broader concerns about governance, accountability, and the legitimacy of the financing model itself (Table 5).

**Table 5:** Cross-tabulations of results for models 7 and 8 (Table 7 complements)

I do not have confidence in the entities responsible for implementing the projects.		
What is the maximum amount you would be willing to pay (in euros, monthly) as a contribution to finance the implementation of projects leading to the mentioned scenario?		Total
Answer = 0	41	41
Total	41	41

Distrust emerges as a decisive determinant of WTP = 0. Among respondents who explicitly selected “I do not have confidence in the entities responsible for implementing the projects,” all 41 reported a willingness to pay of zero. The pattern is absolute: whenever institutional distrust is mentioned, financial refusal is total. This provides clear support for H3b and shows that distrust functions not as hesitation but as a complete psychological and political block to contributing.

The open‑ended justifications reinforce this interpretation. Respondents describe distrust in institutional competence (“there is a lot of money badly spent”), in public management (“taxes should already cover this”, “the State is inefficient”), and in the financing model itself (“I should not be the one to pay”, “companies should contribute”). These statements show that refusal is not primarily economic; it is rooted in institutional, structural, and moral objections to how projects are governed.

The analysis of all reasons for WTP = 0 reveals three distinct patterns. Explicit institutional distrust (41 cases) — always associated with WTP = 0, fully confirming H3b. Operational distrust (23 cases) — respondents believe plans or targets will not be fulfilled, reflecting a lack of credibility in implementation. This reinforces the broader distrust mechanism. Financial constraints (54 cases) — respondents cite inability to pay, representing a practical barrier rather than an attitudinal one.

Taken together, these results show that distrust—explicit or implicit—is strongly associated with total refusal to pay, while financial limitations explain an additional share of zero‑contribution responses. Strengthening institutional credibility, therefore, appears essential for increasing public willingness to support renewable energy investments.

### Stakeholder Involvement

Finally, results for Models 9 and 10 (see Table 7) are to be presented. These allow us to test the set of H4 hypotheses and answer Q1. This time, ordered probit models were used, and the results are presented in Table 6.

**Table 6:** Models 9 and 10 results: Ordered Probit estimations (Table 7 complements)

	(1)	(2)	(3)	(4)	(5)	(6)	(7)	(8)	(9)	(10)
	D19ESP	D19ESP	D19ML	D19ML	D19C	D19C	D19EMP	D19EMP	D19GC	D19GC
main										
D20ERSE	–0.0156	–0.0156								
	(0.0752)	(0.0660)								
D20AA	0.289^***^	0.289^***^								
	(0.0821)	(0.0625)								
D20GL			0.0164	0.0164						
			(0.0614)	(0.0612)						
D20CS					0.0398	0.0398				
					(0.0591)	(0.0641)				
D20E							0.0867	0.0867		
							(0.0624)	(0.0688)		
D20GC									0.00238	0.00238
									(0.0616)	(0.0911)
cut1	–1.040^***^	–1.040^***^	–2.156^***^	–2.156^***^	–1.860^***^	–1.860^***^	–1.736^***^	–1.736^***^	–1.910^***^	–1.910^***^
	(0.324)	(0.190)	(0.302)	(0.340)	(0.260)	(0.238)	(0.255)	(0.166)	(0.264)	(0.358)
cut2	–0.650^**^	–0.650^***^	–1.869^***^	–1.869^***^	–1.224^***^	–1.224^***^	–1.292^***^	–1.292^***^	–1.558^***^	–1.558^***^
	(0.296)	(0.151)	(0.267)	(0.300)	(0.210)	(0.168)	(0.218)	(0.194)	(0.240)	(0.371)
cut3	0.214	0.214	–0.728^***^	–0.728^***^	–0.0849	–0.0849	–0.217	–0.217	–0.565^***^	–0.565^*^
	(0.290)	(0.142)	(0.209)	(0.190)	(0.202)	(0.243)	(0.208)	(0.219)	(0.205)	(0.303)
cut4	1.020^***^	1.020^***^	0.312	0.312	0.632^***^	0.632^***^	0.725^***^	0.725^***^	0.203	0.203
	(0.291)	(0.184)	(0.201)	(0.236)	(0.205)	(0.226)	(0.210)	(0.228)	(0.197)	(0.267)
N	281	281	288	288	289	289	288	288	289	289
Log-likelihood	–333.5	–333.5	–336.7	–336.7	–390.6	–390.6	–372.3	–372.3	–366.1	–366.1
LR chi2	15.32	.	0.0716	0.0719	0.453	0.384	1.928	1.586	0.00149	0.000681
Pseudo R2	0.0289	0.0289	0.000115	0.000115	0.000648	0.000648	0.00285	0.00285	0.00000213	0.00000213
AIC	679.1	677.1	683.4	683.4	791.2	791.2	754.7	754.7	742.3	742.3
BIC	700.9	695.3	701.7	701.7	809.5	809.5	773.0	773.0	760.6	760.6

Standard errors in parentheses. * *p* < 0.10, ** *p* < 0.05, *** *p* < 0.01. See Table 7 for acronyms. Columns (1), (3), (5), (7), and (9) are for robust standard errors. Columns (2), (4), (6), (8), and (10) correspond to clustered robust standard errors. The clustered variable consists of H73CR (What is your municipality of residence? Águeda; Alcanena; Castro Daire; Ovar; Santa Comba Dão; Torres Vedras)

Although the ordered probit models exhibit low explanatory power (Table 6), the results reveal a clear pattern. Across all ten specifications, only one variable—D20AA, trust in Environmental Associations (EAs)—shows a consistent and statistically significant effect. Its positive coefficient indicates that respondents who trust EAs are more likely to perceive the distribution of benefits from renewable energy parks as fair. This effect is robust across both standard and clustered standard errors and stands out as the only meaningful predictor in the full set of models.

By contrast, trust in the Energy Services Regulatory Authority (ERSE), local or central government, civil society organizations, and the media remains statistically insignificant. Their coefficients are small and unstable, suggesting that trust in political or administrative institutions does not shape perceptions of distributive justice in this dataset. While respondents clearly differentiate between fairness categories (as shown by the ordered probit cut‑points), institutional trust explains only a small share of the variation.

Taken together, these results provide partial support for H4. Trust matters, but not uniformly: only trust in a specific regulatory authority—perceived as competent, independent, and credible—affects fairness perceptions. This selective effect aligns with the study’s broader findings: institutional credibility is unevenly distributed, and only certain institutions are trusted enough to influence justice evaluations.

These results also help answer Q1. Trust does influence perceptions of distributive justice, but its impact depends on the institution. Combined with the strong evidence from H3b—where distrust leads to categorical refusal to pay—this suggests that trust is a foundational condition for both distributive and financial support. Regulatory trust appears more influential than political trust, reinforcing the idea that citizens judge fairness not only by outcomes but by the credibility of the institutions overseeing them.

### Discussion of Results

In several cases, especially when Probit and Tobit models show limited statistical significance due to small sample sizes or partial non-response, some relationships should be interpreted as indicative patterns rather than definitive causal effects.

The findings reveal a strong distinction between procedural justice and procedural formality, helping to explain why some governance mechanisms influence acceptance while others do not. Across all descriptive analyses, participation in consultation is consistently linked to more positive perceptions of both procedural and distributive justice: participants feel heard, perceive more community benefits, and view benefit distribution as fair. These relational experiences confer legitimacy and align with the energy‑justice literature, which emphasizes the roles of participation, fairness, and trust (Modica and Rampa, 2026; Ellis and Ferraro, 2016; Gross, 2007; Grelle and Hofmann, 2024; Knauf and Wüstenhagen, 2023; Bourdin, 2026; Aitken, 2010).

In contrast, procedural formality—awareness of EIAs, licensing bodies, or regulatory procedures—shows no effect on acceptance (H2a, H2b). EIA knowledge does not change perceptions of impacts, benefits, or discomfort. This supports critiques that formal procedures often fail to build legitimacy when they are inaccessible or perceived as symbolic (Schnell and Mattes, 2026; Xu et al., 2023; Radtke, 2025; Ellis and Ferraro, 2016; Bourdin, 2026; Aitken, 2010). These findings should not be interpreted as implying that regulatory frameworks or formal procedures lack importance. Robust regulatory procedures remain essential to ensuring transparency, accountability, minimum participation rights, and institutional oversight in renewable energy governance. However, the results suggest that procedural compliance alone may not be sufficient to produce perceived legitimacy or acceptance among affected residents. In other words, formally participatory procedures may fail to generate trust or fairness perceptions if local communities perceive engagement processes as symbolic, inaccessible, or lacking real influence over decisions.

The contrast between participation and procedural formality is particularly important for interpreting the results. Although regulatory procedures such as EIAs formally include consultation requirements, awareness of these procedures does not necessarily imply that residents perceive the process as meaningful, accessible, or influential. The findings, therefore, suggest that institutional compliance with participatory requirements is, by itself, insufficient to generate legitimacy or acceptance. What appears to matter more is whether residents experience engagement as responsive and substantively meaningful rather than merely procedural or administrative.

Perceived impacts (H3a) show a consistent negative association with acceptance, confirming that people respond to concrete expectations about how projects affect daily life (Batel et al., 2013; Wolsink, 2007). General attitudes toward renewable technologies, however, do not reliably predict acceptance.

The strongest evidence concerns trust (H3b). Distrust in responsible institutions is associated with categorical refusal to pay: all respondents expressing distrust reported WTP = 0, an absolute pattern consistent with the “protest zero” mechanism (Sun et al., 2025; Mitchell and Carson, 1981, 1989). Qualitative responses show that distrust reflects concerns about competence, fairness, and moral legitimacy, making trust a threshold condition for economic cooperation (Modica and Rampa, 2026; Sovacool et al., 2022).

Results for H4 show that trust is institution‑specific. Only trust in Environmental Associations increases perceptions of distributive justice, while trust in political institutions does not. This supports research showing that citizens differentiate between institutions based on perceived independence and competence (Knauf and Wüstenhagen, 2023; Ryder et al., 2023; OECD, 2017; Wolsink, 2007).

Overall, the findings support a relational model of acceptance: people judge renewable energy projects based on trust, fairness, participation, and perceived impacts—not on technical knowledge or formal procedures. The study reinforces and extends the energy‑justice framework by showing that meaningful participation strengthens procedural and distributive justice. Also, procedural formality alone (EIA awareness, licensing knowledge) does not influence acceptance. Moreover, distrust leads to complete withdrawal of financial support (WTP = 0). Perceived impacts matter more than general attitudes. Finally, trust effects are selective, with regulatory trust shaping fairness perceptions more than political trust.

The findings are particularly relevant in the Portuguese context, where renewable energy expansion has accelerated under national decarbonization and energy-transition strategies. Although Portugal has made significant progress in renewable electricity generation, recent debates over wind, especially large-scale solar projects, have intensified local concerns about land use, landscape transformation, procedural fairness, and the distribution of benefits. The results suggest that, within this context, formal compliance with regulatory requirements alone is insufficient to generate local legitimacy. Instead, acceptance depends more strongly on institutional credibility, meaningful participation, and the perception that local communities are treated fairly within renewable deployment processes. These findings highlight the importance of strengthening participatory governance practices in Portugal’s ongoing energy transition.

These results empirically validate an integrated institutional pathway to acceptance that links participation, trust, perceived impacts, and fairness within a single analytical framework. However, it is important to clarify that the study does not evaluate or compare different participation formats (e.g., public meetings, online consultations, workshops). The findings, therefore, do not imply that formats are interchangeable or irrelevant. Rather, the results indicate that what influences acceptance is not the specific format used, but whether residents experience the engagement as meaningful, responsive, and influential. This helps explain why participation is associated with higher perceptions of procedural and distributive justice, while procedural formality (e.g., awareness of EIAs or licensing steps) shows no effect. Formal procedures may include participatory requirements, but these do not automatically translate into perceived fairness unless the engagement process, regardless of format, creates genuine opportunities for voice and influence.

This study contributes to the international literature on renewable energy acceptance and energy justice in several ways. First, it advances existing research by disaggregating institutional trust across different stakeholders—including government, regulators, environmental associations, companies, and media actors—showing that trust effects are selective rather than uniform. Second, it demonstrates the distinction between procedural formality and meaningful procedural justice by showing that formal mechanisms such as EIA awareness or regulatory knowledge do not necessarily increase legitimacy or acceptance unless accompanied by credible and participatory governance practices. Third, the study integrates participation, distributive justice, perceived impacts, and willingness-to-pay behavior within a single analytical framework, connecting social acceptance research with the literature on protest responses and institutional legitimacy. Finally, by comparing wind and solar projects in the Portuguese context, the paper extends international scholarship beyond technology-specific case studies. It provides evidence relevant to broader debates on socially legitimate energy transitions.

## Conclusions and policy implications

Public acceptance of renewable energy projects is driven mainly by relational and justice‑based factors, not by technical knowledge or formal regulatory procedures. Procedural justice—feeling included, respected, and treated fairly—consistently predicts more positive attitudes, while awareness of EIAs or regulatory structures has no measurable effect. Formal procedures that lack meaningful engagement remain largely invisible to residents. Formal procedures are necessary but not sufficient. The findings do not suggest that formal regulatory procedures are irrelevant for public acceptance. Rather, they indicate that the existence or awareness of formal procedures alone may be insufficient to generate perceptions of legitimacy, fairness, or acceptance if communities do not experience participation as meaningful and responsive.

Trust is a decisive determinant of support. The willingness‑to‑pay results show an absolute pattern: when trust in responsible institutions collapses, WTP drops to zero. Distrust does not reduce support; it eliminates it, highlighting the central role of institutional credibility.

Perceived impacts also strongly influence acceptance. People respond to concrete expectations about how a project will affect their daily lives, not to general attitudes toward renewable technologies. Institutional credibility is uneven: only trust in an independent, technically competent regulator (the Competition Authority) increases perceptions of distributive justice, while trust in political actors does not.

Overall, acceptance depends on how people are treated, not on how much technical information they receive. Technical procedures influence attitudes only when they are clearly communicated, embedded in participatory processes, and linked to trusted institutions. These findings reinforce the energy‑justice framework, where legitimacy, fairness, and trust are central to the social acceptance of renewable energy transitions.

In the Portuguese context, these findings suggest that renewable energy governance should move beyond compliance-oriented consultation procedures toward more participatory and territorially sensitive approaches. As Portugal continues to expand wind and solar capacity to meet national and European decarbonization objectives, strengthening local legitimacy will become increasingly important to reduce conflict and implementation delays. Policies that improve transparency, reinforce institutional credibility, and ensure visible and equitable local benefits may be particularly important in municipalities directly affected by renewable infrastructure expansion.

Moreover, policy implications include rebuilding institutional trust, ensuring meaningful participation, communicating impacts clearly, making benefit‑sharing transparent, designing credible financial models, and making EIAs visible and understandable—with low variation in EIA knowledge, the cross‑sectional design, and the geographically limited sample.

This study presents several limitations that should be considered when interpreting the findings. First, some estimations rely on relatively small subsamples due to partial non-response in technology-specific questions, reducing statistical power and limiting the robustness of certain comparisons. Second, the cross-sectional design precludes causal inference and captures perceptions at a single point in time. However, attitudes toward renewable energy projects may evolve over the course of project development. Third, the sample is geographically restricted to municipalities in the Centro Region of Portugal, which may limit the generalizability of the results to other institutional and socio-political contexts. In addition, awareness of Environmental Impact Assessments showed limited variation among respondents, constraining the ability to detect statistically significant effects. These limitations suggest caution when extrapolating the findings and highlight the importance of future longitudinal and cross-country comparative research.

These limitations also point to important directions for future research. Comparative studies involving additional Portuguese regions and international case studies would help evaluate the extent to which the relationships identified here depend on specific territorial or institutional conditions. Longitudinal research could further examine how trust, perceptions of fairness, and participation evolve across different phases of renewable energy project development. Future studies could also explore how different governance models and compensation arrangements influence local legitimacy and acceptance in contexts of accelerated renewable energy expansion.

### Ethics Approval

Participation was voluntary. Respondents received clear written/oral information about the study purpose, what participation involved, expected duration, potential risks and benefits, the absence of penalties for non-participation, and their right to withdraw at any time without providing a reason. Informed consent was obtained before the interview started. No sensitive personal data was collected.

## Data Availability

Data sharing is not available due to participant confidentiality restrictions on informed consent; thus, data cannot be shared.

## Appendix

**Table 7:** Variables, Acronyms, Models, Hypotheses, Research Questions, and Methodology

Questions	Hypotheses	Acronym	Dependent	Acronym	Independents	Model	Methodology
Q1 & Q2	H1a & H1b	D23SN	Participated in the public consultation process? (Y/N)	D24SN	You feel that your concerns/interests have been taken into account during the development of the park(s) project in your Municipality? (Y/N)	1	Probit
				D242SNNS	He feels that the distribution of these benefits is fair, for the benefit of the community? (Y/N/DNK)		
				D241SNNS	Have the perception that there are financial benefits for the Municipality/local community? (Y/N/DNK)		
				D22SNNS	Was there a public consultation process regarding the project of the park(s) of your Municipality? (Y/N/DNK)		
		C11SN	Believes wind energy can trigger negative impacts? (Y/N)	D23SN	Participated in the public consultation process? (Y/N)	2	Probit
				D24SN	You feel that your concerns/interests have been taken into account during the development of the park(s) project in your Municipality? (Y/N)		
				D242SNNS	He feels that the distribution of these benefits is fair, for the benefit of the community? (Y/N/DNK)		
				D241SNNS	Have the perception that there are financial benefits for the Municipality/local community? (Y/N/DNK)		
				D22SNNS	Was there a public consultation process regarding the project of the park(s) of your Municipality? (Y/N/DNK)		
				E27SN	Do you feel uncomfortable with the presence of the wind farm? (Y/N)		
				E28SN	Do you feel that the wind farm brings benefits? (Y/N)		
		C12SN	Believes that solar energy can trigger negative impacts? (Y/N)	D23SN	Participated in the public consultation process? (Y/N)	3	Probit
				D24SN	You feel that your concerns/interests have been taken into account during the development of the park(s) project in your Municipality? (Y/N)		
				D242SNNS	Do you feel that the distribution of these benefits is fair for the community? (Y/N/DNK)		
				D241SNNS	Have the perception that there are financial benefits for the Municipality/local community? (Y/N/DNK)		
				D22SNNS	Was there a public consultation process regarding the project of the park(s) of your Municipality? (Y/N/DNK)		
				F38SN	Do you feel uncomfortable with the presence of the solar park? (Y/N)		
				F39SN	Do you feel that the solar park brings benefits? (Y/N)		
	H2a	D21SN	Do you know if the park(s) project in your Municipality has had an environmental impact assessment? (Y/N)	C11SN	Believes wind energy can trigger negative impacts? (Y/N)	4	Probit
				C12SN	Believes that solar energy can trigger negative impacts? (Y/N)		
				E25SN	Get to know the wind farm located in your area? (Y/N)		
				E27SN	Do you feel uncomfortable with the presence of the wind farm? (Y/N)		
				E28SN	Do you feel that the wind farm brings benefits? (Y/N)		
				F36SN	Get to know the solar park located in your area? (Y/N)		
				F38SN	Do you feel uncomfortable with the presence of the solar park? (Y/N)		
				F39SN	Do you feel that the solar park brings benefits? (Y/N)		
Q2	H2b	GF47VMDP	What is the maximum amount you would be willing to pay (in euros, monthly) as a contribution to finance the implementation of renewable energy projects that reduce the price of electricity and CO2 emissions? (Max = 780; Min = 0)	G48PMI	On a scale of 1 to 10, where 1 corresponds to “very little certainty” and 10 to “absolute certainty”, say with what degree of certainty you would be willing to pay	5	Tobit
				C10SN	Do you believe that renewable energy sources for electricity production benefit the population? (Y/N)		
				What is your opinion on how environmentally friendly these energy sources for electricity generation are? (Very friendly=1; Something friend=2; Indifferent=3; Something unfriendly=4; No friend=5; I don’t know=6)			
				C9N	Nuclear		
				C9H	Water		
				C9C	Coal		
				C9GN	Natural gas		
				C9E	Wind		
				C9S	Solar		
				C9GEO	Geothermal		
				C9B	Biomass		
				C9G	Diesel		
				C9EO	Wave Energy		
				Evaluate the importance of each of the following benefits, where 1 means nothing important and 5 means very important?			
				C101I	It is inexhaustible on a human scale		
				C101NPE	Does not produce hazardous emissions or toxic solids		
				C101RC	Reduces contribution to climate change		
				C101FE	Favourable to employment and job creation		
				C101RDE	Reduces external energy dependence on our energy		
Q1	H3a	GF47VMDP	What is the maximum amount you would be willing to pay (in euros, monthly) as a contribution to finance the implementation of renewable energy projects that reduce the price of electricity and CO2 emissions? (Max = 780; Min = 0)	Please rate your level of trust in the following stakeholders. (Very low confidence=1; Low confidence=2; Neutral=3; High Reliability=4; Very high confidence=5; I don’t know=6)		6a & 6b	Tobit
				D20GL	Local government		
				D20CS	Media		
				D20GC	Central government		
				D20ERSE	Energy Services Regulatory Authority		
				D20AA	Environmental associations		
				D20E	Companies		
				How do the following factors contribute to the answer given in question 47 (GF47VMDP)? (1 - not at all important, 5 - very important)			
				G49RE	Reduction of CO2 emissions		
				G49RPE	Reduction in the price of electricity		
				G49IP	Possible impacts in terms of landscape		
				G49IFF	Possible impacts in terms of fauna and flora		
				G49IE	Possible impacts in terms of employment		
				G49IE	Possible impacts in terms of economy		
				G49R	Your current income level		
Q1	H3b	GF47VMDP	What is the maximum amount you would be willing to pay (in euros, monthly) as a contribution to finance the implementation of renewable energy projects that reduce the price of electricity and CO2 emissions? (Max = 780; Min = 0)	If you answered 0 in question 47 (GF47VMDP), please indicate which of the reasons best justify your answer:		7	Tobit
				G50PNSC	I think that the plan would not be fulfilled and, therefore, these forecasts for 2030 would not be verified		
				G50NTC	I don’t have confidence in the entities that would be responsible for implementing the projects		
				G50NVPP	I don’t see any positive points in implementing renewable energy sources		
				G50NTCF	I don’t have the financial capacity to make monetary contributions		
				G50O	Other Answers obtained: I don’t pay what is an obligation of the state to promote, because taxes serve for the state to be active and not a thief I don’t think I should be the one to pay I believe that Taxpayers do not have to pay. It is part of the investor to invest, compete and sell his product, without taxpayer intervention I don’t see why the citizen has to pay for changes to situations created by consumerist needs I don’t believe that there should be new contributions from the population. There are already too many subsidies to polluting companies (fuel companies) that can be redirected Companies can contribute There is a lot of money badly spent by the responsible entities (State, local government, ….). There would be no need for a monetary contribution. Make good use of the existing one.		
Q1	H3a & H3b	GF47VMDP	What is the maximum amount you would be willing to pay (in euros, monthly) as a contribution to finance the implementation of renewable energy projects that reduce the price of electricity and CO2 emissions? (Max = 780; Min = 0)	G52SN	There is some renewable technology (wind, solar, hydro, among others) that should be considered a priority? (Y/N, all are equally relevant)	8	Tobit
				Which one(s)?			
				G53H	Water		
				G53E	Wind		
				G53S	Solar		
				G53EO	Wave energy		
				G53B	Biomass		
				G53G	Geothermal		
				G53O	Other Answer given: Humanity must invest heavily in scientific and technological research that will allow nuclear fusion to be profitable		
Please specify what level of involvement the following stakeholders should have in the wind/solar farm development process, where 1 means no involvement, and 5 means a lot of involvement. Associations for H4 hypotheses: D19ESP & D20ERSE & D20AA; D19ML & D20GL; D19GC & D20GC; D19C & D20CS; D19EMP & D20E							
Q1	H4a	D19ESP	Experts	D20ERSE	Energy Services Regulatory Authority	9a	Oprobit
				D20AA	Environmental associations		
		D19ML	Local municipalities	D20GL	Local government	9b	Oprobit
		D19C	Citizens	D20CS	Media	9c	Oprobit
		D19EMP	Companies	D20E	Companies	9 d	Oprobit
	H4b	D19GC	Central government	D20GC	Central government	10	Oprobit

H1a: Participating in public consultation increases the perception that concerns have been addressed. H1b: Participating increases the perception of distributive justice of benefits. H2a: Knowledge about the existence of EIA improves the acceptance of parks. H2b: Increased energy literacy increases support for new investments. H3a: Trust in decision-makers increases support for new energy projects. H3b: Distrust increases the probability of WTP = 0. H4a: The perception that certain stakeholders should have “high involvement” is associated with the declared level of trust. H4b: Distrust in central government is associated with preferences for greater municipal involvement. Based on the previously defined hypothesis, we aim to answer the following research question(s): Q1) To what extent do perceptions of distributive justice regarding the benefits of parks differ across different stakeholders (local/central government, companies, regulator, NGOs, media)? Q2) Does public participation reduce the perception of negative impacts or increase social acceptance? Q3) Do residents perceive the processes of participation and transparency in wind versus solar projects differently? And Q4) How do energy literacy, available information, and desired engagement in the uptake of new renewable investments vary?

**Table 8:** Estimation of Models 1-4: Probit regressions

	(1)	(2)	(3)	(4)	(5)	(6)	(7)	(8)
	D23SN	D23SN	C11SN	C11SN	C12SN	C12SN	D21SN	D21SN
main								
D24SN	0	0	0	0	–11.31^***^	–11.31^***^		
	(.)	(.)	(.)	(.)	(0.195)	(0.301)		
D242SNNS	0.440	0.440	–0.842	–0.842	–3.352^***^	–3.352^***^		
	(0.761)	(0.574)	(0.690)	(0.655)	(0.326)	(0.230)		
D241SNNS	–0.464	–0.464	0	0	8.924^***^	8.924^***^		
	(0.827)	(0.948)	(.)	(.)	(0.407)	(0.250)		
D22SNNS	0	0	0	0	0	0		
	(.)	(.)	(.)	(.)	(.)	(.)		
D23SN			0.842	0.842	17.92^***^	17.92^***^		
			(1.180)	(0.655)	(0.672)	(0.484)		
E27SN			0	0			0	0
			(.)	(.)			(.)	(.)
E28SN			0	0			0	0
			(.)	(.)			(1.253)	(1.253)
F38SN					3.402^***^	3.402^***^	0	0
					(0.331)	(0.247)	(.)	(.)
F39SN					–14.52^***^	–14.52^***^	0	0
					(0.441)	(0.330)	(.)	(.)
C11SN							0	0
							(.)	(.)
C12SN							0	0
							(.)	(.)
E25SN							0	0
							(.)	(.)
F36SN							0	0
							(.)	(.)
N	20	20	7	7	16	16	2	2
Log-likelihood	–13.69	–13.69	–3.888	–3.888	–2.871	–2.871	–1.386	–1.386
LR chi2	0.338	0.832	1.487	.	.	.	0	0
AIC	31.38	31.38	11.78	9.777	13.74	13.74	4.773	4.773
BIC	33.37	33.37	11.67	9.723	16.83	16.83	3.466	3.466

Standard errors in parentheses. ^*^ *p* < 0.10, ^**^ *p* < 0.05, ^***^ *p* < 0.01. See Table 7 for acronyms. Columns (1), (3), (5), and (7) are for robust standard errors. Columns (2), (4), (6), and (8) correspond to clustered robust standard errors. The clustered variable consists of H73CR (What is your municipality of residence? Águeda; Alcanena; Castro Daire; Ovar; Santa Comba Dão; Torres Vedras)

Table 7, Table 8
